# Far-UVC (222 nm) Enhances the Advanced Reduction Process for Per- and Polyfluoroalkyl Substance (PFAS) Destruction

**DOI:** 10.1021/acsestwater.5c00730

**Published:** 2025-09-12

**Authors:** Xiaoyue Xin, Jiaqi Li, Ching-Hua Huang

**Affiliations:** School of Civil and Environmental Engineering, 1372 Georgia Institute of Technology, Atlanta, Georgia 30332, United States

**Keywords:** hydrated electron, PFAS, PFOS, Far-UVC, KrCl* excimer lamp, advanced reduction process, defluorination, water treatment

## Abstract

The UV-based advanced reduction processes (ARPs) have emerged as an effective strategy to degrade PFAS contaminants in water. This study investigates PFAS degradation by integrating far-UVC irradiation at 222 nm with sulfite-based ARPs. Comparative analysis of UV222/sulfite and conventional UV254/sulfite revealed that UV222/sulfite systems significantly improve the performance by generation of more hydrated electrons (e_aq_^–^), the primary reactive species driving PFAS degradation, and exhibit superior energy efficiency, characterized by lower electrical energy per order (*E*_*EO*_). The higher efficiency of UV222/sulfite can be attributed to stronger light absorption of sulfite and higher photon energy at 222 nm. Under optimized stepwise sulfite dosing conditions, the UV222/sulfite ARP achieved high perfluorooctyl sulfonic acid (PFOS) removal efficiency, nearly 85% reduction in parent compound and 66% defluorination within a 6 h period, while the degradation of shorter-chain PFHxS and PFBS was slower. Real water matrix components can influence treatment efficiency. The impacts of nitrate/nitrite were transient and diminished after rapid photolysis at 222 nm, while dissolved organic matter and carbonates exerted strong reactive species scavenging effects. This study establishes UV222/sulfite ARP as a promising strategy to enhance PFAS degradation. Careful optimization of UV222/sulfite system parameters and water matrices will increase the adaptability for environmental PFAS remediation.

## Graphical abstract

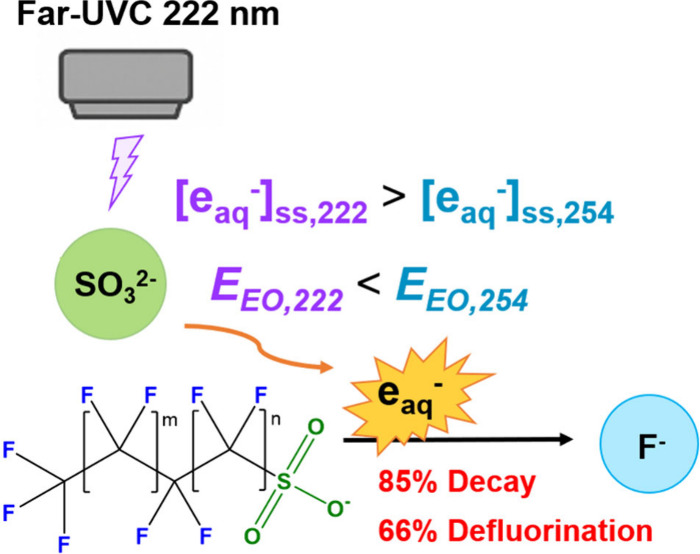

## 1 Introduction

Per- and polyfluoroalkyl substances (PFAS) are a class of anthropogenic organofluorine compounds that have garnered substantial attention due to their environmental persistence, bioaccumulative properties, and potential adverse human health impacts.^1–3^ PFAS have been used in a large variety of industrial and consumer products such as firefighting foams, water-repellent fabrics, carpets, food packaging, and nonstick cookware.^4,5^ The disposal of PFAS-containing products and discharge of contaminated wastewaters result in the omnipresence of these compounds in the environment,^3,6^ tissues of wildlife,^7,8^ and even human blood and breast milks.^3,9–11^ Recognizing the risks associated with PFAS, the U.S. Environmental Protection Agency (EPA) announced the final National Primary Drinking Water Regulation (NPDWR) for six PFAS on April 10, 2024, including perfluorooctanoic acid (PFOA), perfluorooctanesulfonic acid (PFOS), perfluorobutanesulfonic acid (PFBS), perfluorohexanesulfonic acid (PFHxS), perfluorononanoic acid (PFNA) and hexafluoropropylene oxide dimer acid (HFPO–DA or so-called GenX). EPA established the enforceable levels (or maximum contaminant levels, MCLs) in drinking water for five individual PFAS: PFOA and PFOS at 4 ng·L^–1^, respectively; PFHxS, PFNA, and HFPO–DA at 10 ng·L^–1^, respectively; and for PFAS mixtures containing at least two or more of PFHxS, PFNA, HFPO–DA, and PFBS using a hazard index (HI = 1.0) approach.^12^

Most PFAS are highly recalcitrant to conventional water treatment processes,^13–16^ thus, addressing PFAS pollution requires the development of efficient remediation technologies. Among various advanced treatment methods, UV-based advanced reduction processes (UV/ARPs) have emerged as promising strategies to chemically degrade various contaminants primarily by the powerful reducing species hydrated electrons (e_aq_^–^), which are generated through UV photolysis of chemical sensitizers, such as sulfite and iodide.^17–25^ These reductive systems have predominantly been performed using low-pressure UV lamps (LPUV) emitting primarily at 254 nm or medium-pressure UV lamps (MPUV) emitting in the range of 200–400 nm. However, despite their broad applications, conventional UV lamps possess inherent drawbacks, including environmental risks associated with mercury contamination and relatively lower photon energy levels, which may limit their effectiveness for specific advanced photochemical applications. Moreover, UV 254 nm-based ARPs exhibit limited efficiency in degrading PFAS. For example, UV254/sulfite is one of the most extensively studied UV/ARP systems. Sulfite exhibits relatively weak absorption under this irradiation (e.g., ε = 17.6–40 M^–1^·cm^–1^ at pH ∼ 9),^26–29^ which severely restricts the efficiency of e_aq_^–^ generation. Achieving complete or near-complete PFAS removal with UV254/sulfite thus often necessitates prolonged irradiation time, increased chemical dosage, or highly alkaline condition (pH ≥ 12), all of which can significantly raise operational costs and environmental impacts.^24,30–32^

Recently, excimer lamps have emerged as a novel alternative UV source that includes a noble gas-halogen dimer generating UV emission when its excited state returns to the ground state.^33^ The krypton chloride (KrCl*) excimer lamps emit narrowly at 222 nm which falls in the so-called far-UVC range of 200–230 nm.^34^ These lamps offer several significant advantages, including higher photon energy compared to conventional UV lamps, low light absorption of water (ε ∼ 0.001 cm^–1^), the complete absence of toxic mercury, notably reduced potential for harm to human tissues during incidental exposure, and output stability at cold temperatures.^34–37^ The 222 nm irradiation has been demonstrated to be highly effective in inactivating pathogens^38–40^ and degrading some organic pollutants.^41–43^ Previous research has also shown that many PFAS, including perfluorocarboxylic acids (PFCAs), fluorotelomer unsaturated carboxylic acids (FTUCAs), and GenX, are susceptible to direct photolysis by UV irradiation at 222 nm.^44^ Recent studies also highlighted the promising potential of UV222-driven ARPs, demonstrating that these processes provide enhanced efficiency in photoreductive degradation and dehalogenation for halogenated organic pollutants.^45,46^ Specifically, ARPs utilizing UV 222 nm irradiation show lower energy consumption and improved contaminant removal performance compared to conventional 254 nm-based ARPs. Nevertheless, despite these encouraging findings, the specific application and efficacy of 222 nm radiation in enhancing ARPs for PFAS destruction remain inadequately studied. Given the inherently higher photon energy associated with the 222 nm emission, the excimer lamps hold the potential to significantly enhance the photochemical production of hydrated electrons from chemical sensitizers. Consequently, these lamps may offer superior PFAS degradation efficiency relative to conventional UV sources, representing a crucial advancement toward more sustainable, efficient, and safer remediation.

This study aims to systematically evaluate UV222/sulfite as an efficient ARP system for the degradation and defluorination of PFAS. First, the fundamental photochemical properties of the UV222/sulfite system were explored by adapting a published *R*_*e–,UV*_ method (defined as the e_aq_^–^ exposure per UV fluence, which was previously used for the UV254/sulfite system),^47^ to quantify and optimize the generation of e_aq_^–^ under 222 nm irradiation. Then, PFOS was selected for an in-depth investigation of the effects of reaction conditions, including varying sulfite dosages and real water matrices. Comparison with LPUV was also conducted for e_aq_^–^ generation and PFOS degradation. Finally, the degradation and defluorination potential of two other regulated PFAS, PFHxS and PFBS, by UV222/sulfite were also evaluated, given their known resistance to treatment by UV-based processes.

## 2 Materials and Methods

### 2.1 Chemicals

Information on chemicals and the preparation of reaction solutions and PFAS stock solutions is provided in SI Text S1. Reagent-grade deionized (DI) water (18.2 mΩ-cm) was generated from a Milli-Q Nanopure water purification system (Billerica, MA).

### 2.2 Experimental Setup

All the UV222 and UV254 irradiation experiments were conducted using a sealed quartz reactor (50 mL reaction solution) placed directly under a light source of a KrCl* excimer lamp (Ushio) or low-pressure Hg lamps (G4T5 Hg lamp, Philips TUV4W) as illustrated in Figure S1. Iodide-iodate actinometry was applied to measure the UV fluence rate received in the reaction solution, which has been proven to be accurate for both 222 and 254 nm irradiation.^48,49^ The UV fluence rate received in the reaction solution was measured to be 5.53 × 10^–7^ Einstein·L^–1^·s^–1^ for UV222, and 3.10 × 10^–7^ Einstein·L^–1^·s^–1^ for UV254, respectively. The reaction solution volume was 50 mL, and the effective path length was 2.35 cm. All experiments were conducted at room temperature (20 °C).

Given that previous studies have indicated alkaline conditions (e.g., pH > 9) effectively minimize the scavenging of hydrated electrons (e_aq_^–^) by acidic protons (H^+^),^19,29^ a solution pH of 10.0, maintained using 1.0 mM borate buffer, was selected for this study to ensure efficient e_aq_^–^ generation. All reaction solutions were first purged by nitrogen gas for 1 h to minimize the dissolved oxygen interference. Concentrated stock solutions of sodium sulfite, monochloroacetic acid (MCAA), or selected PFAS were then spiked into the reactors and allowed to mix for at least 1 min. The reactor was then positioned under the selected lamp(s), and aliquots of solution (0.3 mL) were collected at specific time intervals using a syringe. Samples for anion analysis were collected in 2 mL amber glass vials and analyzed within 12 h. Samples for PFAS analysis were stored in 2 mL amber polypropylene vials at 4 °C before analysis.

### 2.3 Analytical Methods

Extracted samples containing PFAS were injected to an Agilent 1260 Infinity high performance liquid chromatography (HPLC) with 6410 Triple Quad LC-MS/MS system under electrospray ionization in negative (ESI^–^) ion mode. The MS was operated in multiple reaction monitoring (MRM) acquisition mode, selecting two transitions (if possible) for each analyte and internal standards (IS), as reported in Table S1. MS parameter setting was optimized by the individual infusion of neat standards and by ramping cone voltage and collision energy. The drying gas flow rate was 6 L·min^–1^, gas temperature 350 °C, nebulizer pressure 40 psi, and capillary voltage 3500 V. Sample injection volumes were 20.0 μL. A Poroshell 120 EC-C18 column (2.1 × 150 mm, 4 μm) was maintained at 35 °C, using chromatographic method consisting of a multistep gradient lasting 23.5 min at a constant 0.25 mL·min^–1^ eluent flow rate with 5.0 mM ammonium acetate and 80/20 (v/v) methanol/acetonitrile. After 2 min hold at 100% of 5.0 mM ammonium acetate, the organic phase was ramped to 70% over 2 min, to 98% over 12 min, followed by a 2 min hold at 98%. The gradient was ramped to 100% of 5.0 mM ammonium acetate, followed by a 5 min postrun equilibration period. An instrument blank consisting of 80/20 (v/v) MeOH/H_2_O was analyzed after the injection of the highest calibration standard to check the potential carryover between injections. Instrument sensitivity was checked by injecting the calibration standard prior to analysis and at least once every 24 h.

Concentrations of MCAA, halide ions (F^–^, Cl^–^), and inorganic anions (SO_3_^2–^, NO_3_^–^, and NO_2_^–^) were analyzed using ion chromatography. A Dionex ICS-3000 ion chromatography (IC) system equipped with a conductivity detector, a Dionex IonPac AS14A column (4 × 250 mm), a Dionex IonPac AG14A (4 × 50 mm) guard column, and a Dionex ADRS 600 (4 mm) suppressor were used in this study. The column was operated at 20 °C with a carbonate/bicarbonate eluent at 0.7 mL·min^–1^ flow rate and a 36-mA suppressor current. Sulfite concentration was double confirmed by the measurement of the absorbance of thiol after reaction with 5,5′-dithiobis­(2-nitrobenzoic acid) (DTNB).^50^

## 3 Results and Discussion

### 3.1 Quantifying Hydrated Electrons in UV/Sulfite ARP Systems

#### 3.1.1 Generation of Hydrated Electrons in the UV222/Sulfite System

The quantification of hydrated electrons (e_aq_^–^) generated in the UV222/sulfite system was performed by adapting a published *R*_*e‑,UV*_ method (defined as the e_aq_^–^ exposure per UV fluence, details in SI Text S2) using monochloroacetic acid (MCAA) as a probe compound.^47^ Under UV222 irradiation, notable direct photolysis of MCAA occurred even without sulfite addition, characterized by a measured pseudo-first-order decay constant (*k*_*obs*_) of approximately 1.90 × 10^–4^ s^–1^, converting into a fluence-based rate constant (*k*_*F*_) of 3.45 × 10^2^ L·Einstein^–1^ (Figure 1(a), Table 1). This direct photolysis highlights the inherent photoreactivity of MCAA at shorter wavelengths, necessitating correction when assessing e_aq_^–^ generation specifically induced by sulfite photolysis.

**Figure 1:**
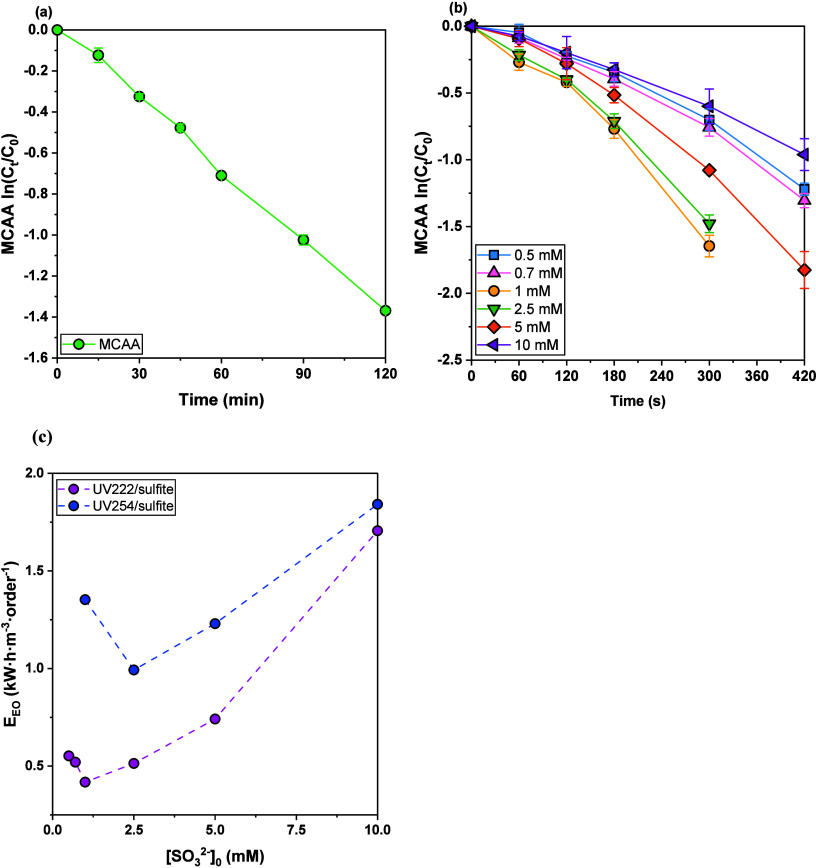
(a) Photodegradation of MCAA under 222 nm irradiation. Reaction conditions: [MCAA]_0_ = 45 μM, [borate] = 1.0 mM, pH = 10. (b) Decay of MCAA by UV222/sulfite. (c) *E_EO_* (kW·h·m^–3^·order^–1^) of UV/sulfite ARP systems under 222 and 254 nm irradiation at varying sulfite dose (mM). Reaction conditions: [MCAA]_0_ = 45 μM, [SO_3_^2–^]_0_ = 0.5–10.0 mM, [borate] = 1.0 mM, pH = 10. Details of *E*_*EO*_ calculation in SI Text S4.

**Table 1:** MCAA Kinetic Data, Calculated *R*_*e‑,UV*_, and [e_aq_^–^]_ss_ ^a^

Sulfite dose (mM)	Time-based *k*_*obs*_ (s^–1^)	Adjusted *k*_*adj*_ (s^–1^)^a^	Fluence-based *k*_*F*_ (L·Einstein^–^)^b^	*R*_*e‑,UV*_ (M·s·L·Einstein^–1^)	[e_aq_^‑^]_ss_ (M)
UV222 (*I*_0_ = 5.53 × 10^–7^ Einstein·L^–1^·s^–1^)					
0	(1.90 ± 0.12) × 10^–4^	/	3.45 × 10^2^	/	/
0.5	(3.76 ± 0.45) × 10^–3^	3.71 × 10^–3^	6.71 × 10^3^	6.71 × 10^–6^	3.71 × 10^–12^
0.7	(4.09 ± 0.48) × 10^–3^	4.05 × 10^–3^	7.33 × 10^3^	7.33 × 10^–6^	4.05 × 10^–12^
1.0	(4.98 ± 0.36) × 10^–3^	4.95 × 10^–3^	8.96 × 10^3^	8.96 × 10^–6^	4.95 × 10^–12^
2.5	(4.51 ± 0.30) × 10^–3^	4.50 × 10^–3^	8.14 × 10^3^	8.14 × 10^–6^	4.50 × 10^–12^
5.0	(3.86 ± 0.30) × 10^–3^	3.85 × 10^–3^	6.97 × 10^3^	6.97 × 10^–6^	3.85 × 10^–12^
10.0	(2.32 ± 0.09) × 10^–3^	2.32 × 10^–3^	4.19 × 10^3^	4.19 × 10^–6^	2.32 × 10^–12^
UV254 (*I*_0_ = 3.10 × 10^–7^ Einstein·L^–1^·s^–1^)					
1.0	(2.97 ± 0.06) × 10^–4^	/	9.57 × 10^2^	9.57 × 10^–7^	2.97 × 10^–13^
2.5	(5.99 ± 0.15) × 10^–4^	/	1.93 × 10^3^	1.93 × 10^–6^	5.99 × 10^–13^
5.0	(7.32 ± 0.12) × 10^–4^	/	2.36 × 10^3^	2.36 × 10^–6^	7.32 × 10^–13^
10.0	(8.16 ± 0.07) × 10^–4^	/	2.63 × 10^3^	2.63 × 10^–6^	8.16 × 10^–13^

a Reaction conditions: [MCAA]_0_ = 45 μM, [borate] = 1 mM, pH = 10, solution was initially purged by N_2_ gas for 1 h.

b *k*_*adj*_ was obtained by subtracting the direct photolysis rate constant of MCAA (*k*_*d*_′) from the observed rate constant (*k*_*obs*_): *k*_*adj*_ = *k*_*obs*_ – *k*_*d*_′ (SI Text S2).

c Fluence-based *k*_*F*_ was calculated based on adjusted *k*_*adj*_.

Upon sulfite addition, a substantial increase in the fluence-based rate constants (*k*_*F*_) was observed, clearly indicating enhanced generation of e_aq_^–^ (Figure 1(b), Table 1). The fluence-based rate constant initially increased significantly from 6.71 × 10^3^ L·Einstein^–1^ at 0.5 mM sulfite to a peak of 8.96 × 10^3^ L·Einstein^–1^ at 1.0 mM sulfite, followed by a progressive decline at higher sulfite concentrations (4.19 × 10^3^ L·Einstein^–1^ at 10 mM). Correspondingly, the calculated hydrated electron exposure per UV fluence (*R*_*e‑,UV*_) reached a maximum value of 8.96 × 10^–6^ M·s·L·Einstein^–1^ at 1.0 mM sulfite, accompanied by a peak steady-state e_aq_^–^ concentration ([e_aq_^–^]_ss_) of approximately 4.95 × 10^–12^ M. The observed decrease in e_aq_^–^ production at sulfite concentrations exceeding 1.0 mM under 222 nm irradiation is primarily attributed to the high molar absorptivity of sulfite at this wavelength (1363.3 M^–1^·cm^–1^). A further increase in sulfite concentration would not enhance sulfite photoactivation due to light absorption saturation. Elevated sulfite levels could continuously limit photon penetration into the solution, as well as promote self-scavenging and localized radical recombination (eqs 1–3),^51^ thereby reducing the effectiveness of e_aq_^–^ generation.

1 $${SO}_{3}^{•-}+SO_{3}^{•-}\to S_{2}O_{6}^{2-}$$

2 $$SO_{3}^{•-}+SO_{3}^{•-}\to SO_{3}^{2-}+SO_{3}$$

3 $$SO_{3}+H_{2}O\to 2H^{+}+SO_{4}^{2-}$$

Therefore, 1.0 mM sulfite is identified as the optimal dosing concentration under UV222, balancing photon absorption efficiency and hydrated electron production.

#### 3.1.2 Comparison between UV222/Sulfite and UV254/Sulfite

In comparison with the conventional UV254/sulfite ARP system, the UV222/sulfite system demonstrated significantly superior e_aq_^–^ production efficiency (Table 1). Under analogous experimental conditions, the maximum *k*_*F*_, *R*_*e‑,UV*_, and [e_aq_^–^]_ss_ for UV254 at 10 mM sulfite were only 2.63 × 10^3^ L·Einstein^–1^, 2.63 × 10^–6^ M·s·L·Einstein^–1^, and 8.16 × 10^–13^ M, respectively, which were approximately 1 order of magnitude lower than the optimal UV222 values. The lower e_aq_^–^ yield with UV254 reflects the much lower molar absorptivity of sulfite at 254 nm (ε_SO32‑,254_ = 22.3 M^–1^·cm^–1^ vs ε_SO32‑,222_ = 1363.3 M^–1^·cm^–1^) and the relatively lower photon energy (472 kJ·mol^–1^ vs 539 kJ·mol^–1^), underscoring the advantage of employing far-UVC 222 nm excimer lamps for more efficient ARP systems.

Based on the MCAA degradation rates at varying sulfite doses, the effective quantum efficiency (Φ_obs_, mol·Ein^–1^) of the UV/sulfite system, defined as the moles of e_aq_^–^ formed divided by the moles of photons absorbed by sulfite, was calculated (details in SI Text S3, Table S3). The averaged Φ_obs_ value was 0.133 mol·Ein^–1^ for the UV222/sulfite system and 0.129 mol·Ein^–1^ for the UV254/sulfite system. A previous study reported an innate effective quantum yield (Φ_innate_ = 0.116 mol·Ein^–1^) for UV254/sulfite, determined by quantifying e_aq_^–^ production under consistent oxygen-free (N_2_-purged) conditions and independently validated with multiple probes (e.g., MCAA, nitrite).^28^ The slightly elevated Φ_obs_254_ value in our study is likely due to additional minor reaction pathways for radicals or methodological differences, such as variations in reactor geometry or photon utilization efficiency. Overall, these results clearly highlight the potential of UV222/sulfite to effectively enhance hydrated electron production, offering advantages for the remediation of recalcitrant contaminants.

The electrical energy per order (*E*_*EO*_, kW**·**h**·**m^–3^ **·**order^–1^) values of UV/sulfite ARP systems under 222 and 254 nm irradiation, respectively, were calculated for both lamp energy (*E*_*EO,UV*_) and chemical reagent costs (*E*_*EO,sulfite*_) (SI Text S4, Table S4). The estimated total *E*_*EO*_ reflects the energy demand for the same amount of hydrated electron generation by the UV/sulfite ARPs. The UV222/sulfite system consistently exhibited lower *E*_*EO*_ than the UV254/sulfite system across the tested sulfite doses (0.5–10 mM) (Figure 1(c)). The 222 nm based ARP achieved a minimum total *E*_*EO*_ of 0.42 kW**·**h**·**m^–3^ **·**order^–1^ at the optimal sulfite dose of 1 mM, nearly half the lowest *E*_*EO*_ attained by the 254 nm based ARP (0.99 kW**·**h**·**m^–3^ **·**order^–1^ at 2.5 mM). This superior efficiency at 1 mM correlated with the highest hydrated electron generation in the UV222/sulfite system, which could lead to maximal contaminant degradation per unit energy. Under 222 nm irradiation, raising sulfite concentration from 0.5 to 1.0 mM sharply lowered the total *E*_*EO*_ from 0.55 kW**·**h**·**m^–3^ **·**order^–1^ to 0.42 kW**·**h**·**m^–3^ **·**order^–1^, indicating improved treatment efficacy as more hydrated electrons were generated to drive pollutant reduction. Beyond this optimum, however, the *E*_*EO*_ rose again (up to 1.71 kW**·**h**·**m^–3^ **·**order^–1^ at 10 mM) as excessive sulfite imposed a high chemical burden and introduced light absorption saturation and self-scavenging effects. Therefore, past the ideal radical generation point, additional sulfite provided diminishing returns in energy efficiency. By contrast, the UV254/sulfite system required a higher sulfite dose to minimize energy cost. At 1 mM, it gave a relatively high *E*_*EO*_ (1.35 kW**·**h**·**m^–3^ **·**order^–1^) due to limited generation of e_aq_^–^ at 254 nm. Increasing to 2.5 mM improved performance (0.99 kW**·**h**·**m^–3^ **·**order^–1^) by producing more e_aq_^–^, but beyond 2.5 mM the total *E*_*EO*_ climbed again (reaching 1.84 kW**·**h**·**m^–3^ **·**order^–1^ at 10 mM), as the added chemical energy outweighed the slight reduction in UV energy demand. Overall, the UV222/sulfite system is more energy-efficient, achieving optimal pollutant removal at a lower reagent dosage. In practice, this translates to lower chemical consumption and electrical energy used for the 222 nm based ARP system, whereas the conventional 254 nm based system entails higher chemical dose requirement as well as greater operational cost.

### 3.2 PFOS Degradation by UV/Sulfite ARP Systems

#### 3.2.1 Comparison between UV222/Sulfite and UV254/Sulfite

To elucidate the influence of irradiation wavelength on the degradation of PFOS, comparative experiments were performed using UV222 and UV254 with the same dosage of sulfite (10 mM) at an equivalent photon fluence (∼4.0 × 10^–3^ Einstein·L^–1^) (SI Figure S2, Table S5). The efficiency of PFOS degradation was assessed through two critical parameters: the overall decay of PFOS concentration and the degree of defluorination (deF%). The overall decay of PFOS was quantified based on C_t_/C_0_. Based on the concentration of fluoride ion released from the PFOS molecules into the solution, the overall defluorination ratio (deF%) was calculated by eq 4:

4 $$deF\%=\frac{C_{F^{-}}}{n\times C_{0}}$$

where *C*_*F* ^–^_ is the molar concentration of fluoride ion released in solution, *C*_0_ is the initial molar concentration of the parent PFAS, and *n* is the number of fluorine atoms in the parent PFAS molecule. Under similar photon fluence, the UV222/sulfite system exhibited a notably higher PFOS decay of 41.3% compared to 35.8% observed for the UV254/sulfite system. Moreover, defluorination, indicative of complete cleavage of C–F bonds and subsequent formation of fluoride ions, was greater under UV222 (26.2%) compared to UV254 (18.8%). Given that direct photolysis of PFOS is negligible under 222 nm irradiation,^44^ the observed enhancement in PFOS degradation and defluorination is primarily attributed to the significantly higher generation of e_aq_^–^ from UV222/sulfite. As discussed in previous section, the UV222/sulfite system demonstrated substantially higher *R*_*e‑,UV*_ and [e_aq_^–^]_ss_ compared to the UV254/sulfite system, indicating a greater availability of e_aq_^–^ for attacking the robust C–F bonds within PFOS.

#### 3.2.2 Optimal Sulfite Dosage for the UV222/Sulfite System

During the UV222/sulfite ARP aimed at degrading PFOS, sulfite consumption emerged as a critical factor influencing the reaction performance. Initial tests demonstrated rapid sulfite depletion within the reaction solution, correlating with diminished PFOS degradation efficiency over time (SI Table S2, Figure S2). To overcome this limitation and sustain the reaction’s effectiveness, a strategy of multiple-portion sulfite dosing was implemented, adding sulfite hourly to maintain target concentrations consistently throughout the 4-h treatment period. Experiments conducted across various target sulfite concentrations ([SO_3_^2–^]_t_ = 0.5–10 mM) revealed significant differences in both PFOS decay and defluorination efficiencies (Figure 2, SI Table S5). At lower sulfite concentrations (0.5 and 0.7 mM), relatively modest PFOS degradation was observed (38.4% and 47.1%, respectively), accompanied by limited defluorination (21.2% and 23.1%, respectively). Increasing the sulfite concentration to 1.0 mM dramatically enhanced performance, achieving optimal PFOS decay (71.8%) and defluorination efficiency (49.8%). This marked improvement reflects an optimal balance where sufficient e_aq_^–^ are continuously generated without excessive scavenging effects. However, further elevating the sulfite concentration beyond 1.0 mM led to diminished returns. For instance, at 2.5 mM, PFOS decay plateaued at 71.8%, while defluorination slightly decreased to 44.3%. Even higher sulfite concentrations (5.0 and 10.0 mM) resulted in notable declines in both PFOS decay (64.2% and 59.0%, respectively) and defluorination efficiencies (41.3% and 31.2%). This suggested that excessive sulfite dosages would affect the generation of e_aq_^–^, thereby limiting the degradation of PFOS. The identified optimal sulfite dosing (1.0 mM) aligns well with previous findings on the quantification of e_aq_^–^ probed by MCAA. Overall, the multiple-portion dosing strategy effectively mitigates sulfite depletion, significantly enhancing the efficiency of UV222/sulfite for PFAS remediation.

**Figure 2:**
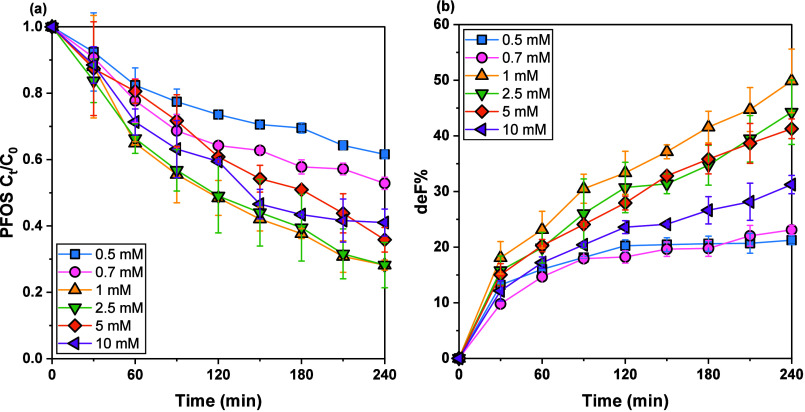
PFOS transformation within 4 h in the UV222/sulfite systems. (a) PFOS decay and (b) defluorination as a function of time. Reaction conditions: [PFOS]_0_ = 8 μM, [borate] = 1.0 mM, pH = 10; sulfite was spiked into water at time 0 and added every hour to maintain a concentration of 0.5–10 mM.

#### 3.2.3 Degradation Products

Degradation products were analyzed from the reactions of PFOS that exhibited significant decay and defluorination under the optimized reaction conditions by UV222/sulfite. Analytical screening revealed several prominent intermediates, notably species corresponding to sequential H/F substitutions on the original eight-carbon PFOS backbone. Specifically, intermediates bearing one (C_8_F_16_H–SO_3_^–^), two (C_8_F_15_H_2_–SO_3_^–^), and three (C_8_F_14_H_3_–SO_3_^–^) hydrogen substitutions were detected in substantial abundance (SI Figure S3­(a)). These observations strongly indicate that the primary degradation pathway under UV222/sulfite ARP conditions involves successive reductive defluorination via H/F exchange without significant shortening of the PFOS carbon chain. This H/F exchange pathway is likely to happen beginning with relatively weak C–F bonds, which mainly occur in the middle of the PFOS structures.^24^ Meanwhile, a series of PFCAs (from PFOA to PFHxA) were also observed with very low intensities. The low initial concentrations of these PFCAs at time zero likely originated as impurities in the commercial PFOS reagent. Although previous studies have demonstrated substantial degradation of PFCAs via UV/sulfite ARPs, the concentration of PFOA observed here did not significantly decrease over time, which implies continuous formation of PFOA from the cleavage of the C–S bond in PFOS under UV222/sulfite conditions. Additionally, the slight increase in shorter-chain compounds such as PFHpA and PFHxA likely results from sequential chain-shortening reactions initiated from PFOA. The likely major degradation mechanism of perfluorosulfonic acids (PFSAs) by UV222/sulfite is summarized in SI Figure S3­(b), based on the experimentally observed PFOS decay and defluorination, and degradation product analyses.

### 3.3 Degradation Performance of Other PFSAs by UV222/Sulfite

In addition to PFOS (C8), the effectiveness of UV222/sulfite in degrading and defluorinating other shorter-chain PFSAs, PFHxS (C6) and PFBS (C4), was also evaluated (Figure 3). Under optimized conditions, where sulfite concentration was maintained consistently at approximately 1 mM through a multiple-portion dosing approach, significant differences in degradation efficiency and defluorination extent were observed among these PFAS, correlating clearly with their molecular structures and chain lengths. As discussed before, PFOS exhibited high susceptibility to degradation and defluorination under UV222/sulfite optimized conditions, achieving 85.2% decay and 66.4% defluorination within a 6-h reaction period (SI Table S5). Comparatively, PFHxS showed moderate reactivity, with overall degradation and defluorination efficiencies reaching 73.2% and 33.5%, respectively, within a longer, 10-h reaction period. The degradation of PFBS was more notably limited, with only 18.4% overall decay and a mere 8.1% defluorination achieved over the same 10-h duration. These results highlight a clear trend that shorter-chain PFAS exhibit lower degradation efficiency and greater recalcitrance toward electron-driven reductive degradation processes.

**Figure 3:**
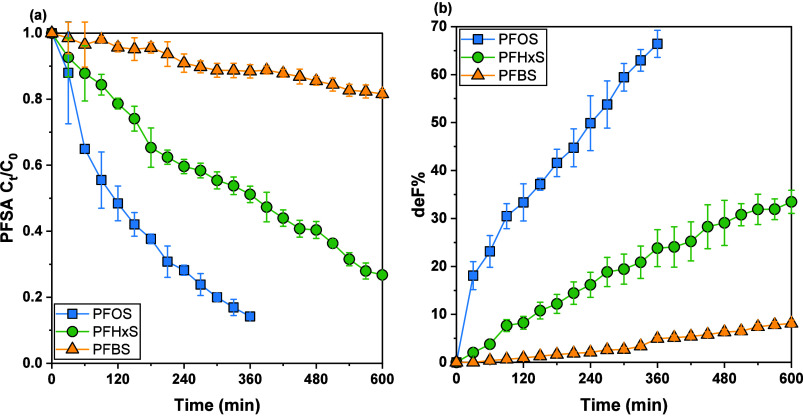
Transformation of selected PFAS (PFOS, PFHxS, and PFBS) in the UV222/sulfite systems. (a) PFAS decay and (b) defluorination as a function of time. Reaction conditions: [PFAS]_0_ = 8 ± 1.7 μM, [borate] = 1.0 mM, pH = 10; sulfite was spiked into water at time 0 and every hour to maintain a concentration of around 1 mM.

Under optimized reaction conditions ([SO_3_^2–^]_t_ = 1.0 mM), the steady-state concentration of hydrated electrons ([e_aq_^–^]_ss_) was determined to be around 4.95 × 10^–12^ M (Table 1). Correspondingly, the calculated second-order reaction rate constants with e_aq_^–^ were 1.83 × 10^7^ M^–1^·s^–1^ for PFOS, 6.71 × 10^6^ M^–1^·s^–1^ for PFHxS, and 1.21 × 10^6^ M^–1^·s^–1^ for PFBS, respectively. These measured rate constants align closely with those previously reported values in literature (1.21 × 10^7^ to 7.30 × 10^7^ M^–1^·s^–1^ for PFOS,^30,47,52^ 6.53 × 10^6^ M^–1^·s^–1^ for PFHxS,^30^ and 1.83 × 10^6^ M^–1^·s^–1^ for PFBS^30^). These results indicate that the UV222/sulfite ARP offers substantial promise for remediating long-chain PFAS, such as PFOS. However, the diminished performance observed for shorter-chain PFAS emphasizes the need for further optimization, potentially through strategies aimed at enhancing electron availability and extending effective reaction time. Addressing the inherent challenges associated with shorter-chain PFAS remains critical, particularly in light of recent regulatory developments such as the U.S. EPA’s enforceable maximum contaminant levels (MCLs). Enhancing the capability of UV/sulfite remediation technologies to effectively degrade short-chain PFAS directly aligns with these stringent regulatory standards, significantly broadening their practical applicability and environmental significance.

### 3.4 Influence of Water Matrices

The performance of UV222/sulfite was assessed for PFOS in various water matrices, including the presence of nitrate (NO_3_^–^), dissolved organic matter (DOM), and dissolved carbonate (Figure 4 and SI Table S5).

**Figure 4:**
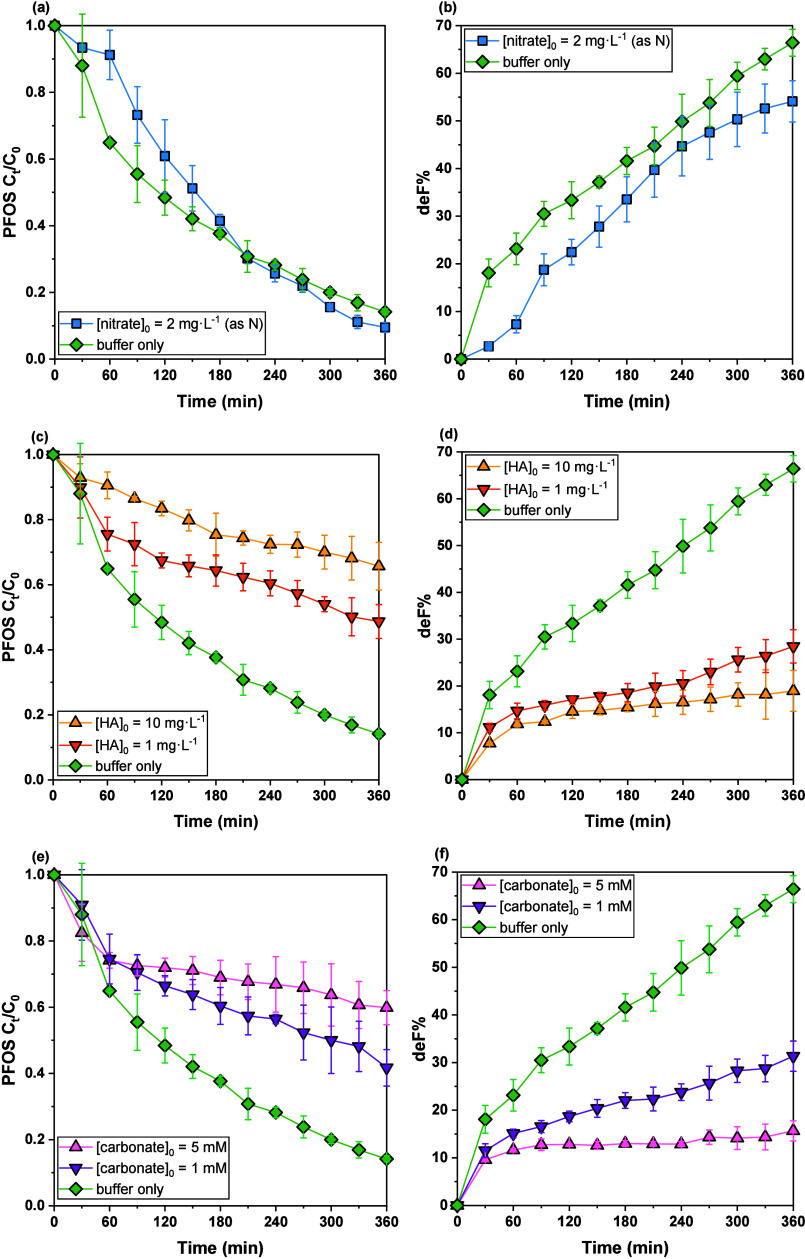
PFOS decay and defluorination within 6 h in the UV222/sulfite systems: (a,b) presence of nitrate, [nitrate]_0_ = 2 mg·L^–1^ (as N); (c,d) presence of NOMs, [HA]_0_ = 1, 10 mg·L^–1^; (e,f) presence of dissolved carbonate/bicarbonate, [HCO_3_^–^ + CO_3_^2–^] = 1, 5 mM. Reaction conditions: [PFOS]_0_ = 8 μM, [borate] = 1.0 mM, pH = 10; sulfite was spiked into water at time 0 and added every hour to maintain a concentration of around 1 mM.

With the presence of nitrate in the UV222/sulfite ARP, PFOS degradation was initially inhibited relative to nitrate-free conditions (Figure 4(a,b)). Nitrate’s high molar absorptivity at 222 nm (ε ∼ 2.8 × 10^3^ M^–1^·cm^–1^) makes it a strong photon scavenger, reducing sulfite photolysis. Moreover, nitrate is a strong e_aq_^–^ scavenger with a bimolecular rate constant *k* = 9.7 ×  10^9^ M^–1^·s^–1^.^29^ Instead of attacking PFOS, e_aq_^–^ preferentially reduces nitrate to nitrite, and the nitrite also absorbs light strongly at 222 nm (ε ∼ 3.3 × 10^3^ M^–1^·cm^–1^) and rapidly reacts with e_aq_^–^ at *k* = 3.5 ×  10^9^ M^–1^·s^–1^,^29^ further prolonging the initial lag phase. Consequently, PFOS decay proceeded more slowly during the first ∼ 1.5 h with nitrate/nitrite present (SI Figure S4). Despite this early inhibition, once nitrate was fully converted and nitrite became undetectable, PFOS degradation resumed and eventually achieved slightly greater removal than the nitrate-free control after 6 h (90.5% vs 85.2% PFOS overall decay). These results suggested transient indirect pathways (possibly involving reactive nitrogen species) modestly enhanced PFOS transformation, offsetting the loss of some reducing power to nitrate. However, defluorination of PFOS was lower with nitrate (54.1% vs 66.4%), indicating nitrate’s presence led to less formation of e_aq_^–^ in the system to break down C–F bonds. In summary, nitrate initially suppresses PFOS degradation in the UV222/sulfite ARP by sequestering UV photons and e_aq_^–^, and while its eventual reduction may slightly improve overall PFOS removal, it compromises defluorination efficiency.

Dissolved organic matter (DOM), modeled by humic acids (HA), significantly impacted the efficiency of PFOS degradation and defluorination in UV222/sulfite (Figure 4(c,d)). The presence of HA greatly inhibited PFOS degradation, with efficiency decreasing as HA concentration increased. Specifically, PFOS degradation decreased from 85.2% (without HA) to 51.3% and 34.3% at HA concentrations of 1 mg·L^–1^ and 10 mg·L^–1^, respectively, over the 6-h irradiation period. Correspondingly, defluorination efficiency also declined substantially, from 66.4% without HA to 28.5% at 1 mg·L^–1^ HA and further to 19.0% at 10 mg·L^–1^ HA. DOM are known to efficiently capture hydrated electrons with the rate constants ranging from 0.51 to 2.11 ×  10^8^ M_C_^–1^·s^–1^.^53^ Consequently, even a modest DOM concentration (1 mg·L^–1^) rapidly captures a large fraction of e_aq_^–^ generated in the UV222/sulfite system, severely limiting their availability for PFOS, thus directly limiting both degradation and defluorination pathways. As the HA concentration increased from 1 to 10 mg·L^–1^, the incremental inhibitory effect became smaller because the majority of e_aq_^–^ had already been scavenged at the lower HA concentration. Although HA can also absorb light under 222 nm irradiation (ε_HA,222_ = 0.047 L·mgC^–1^·cm^–1^),^54^ the light-screening effect of HA at the studied concentrations was minimal compared to the significant absorption by sulfite (1 mM, ε_SO32‑,222_ = 1363.3 M^–1^·cm^–1^). Therefore, the observed inhibitory effects on PFOS removal are predominantly due to HA scavenging of reactive species rather than light-shielding. The observed inverse correlation between HA concentration and PFOS removal rates underscores the critical role of DOM concentration in controlling ARP efficiency. These results suggest that optimizing the UV222/sulfite ARP for real water matrices must consider DOM content. Mitigation strategies, such as DOM pretreatment or system adjustments like increased sulfite dosing or prolonged irradiation, may be required to maintain effective PFOS removal in waters rich in organic matter.

The presence of dissolved carbonate could also significantly influence the performance of UV222/sulfite for PFOS degradation and defluorination. Results demonstrated that increasing total dissolved carbonate concentration notably decreased the efficiency of both PFOS degradation and defluorination (Figure 4(e,f)). Specifically, the PFOS decay rate declined from 85.2% in carbonate-free conditions to 58.3% with 1 mM carbonate, and further diminished to 40.1% at 5 mM carbonate after a 6-h reaction period; while the defluorination rate was significantly reduced from 66.4% in the absence of carbonate to 31.3% at 1 mM carbonate and further to 15.7% at 5 mM carbonate. This inhibitory effect can be primarily attributed to carbonate’s strong scavenging behavior toward e_aq_^–^. Carbonate reacts with e_aq_^–^ with a reported second-order rate constant of approximately 3.9 × 10^5^ M^–1^·s^–1^,^29^ thereby substantially reducing the availability of reactive electrons for PFOS degradation under the tested concentrations. These results underscore the substantial influence of carbonate concentration in real water matrices on UV222/sulfite ARP effectiveness. To optimize PFOS removal in carbonate-rich waters, strategies such as enhancing sulfite dosage or prolonging exposure time may be required. Such considerations are crucial for practical applications and demonstrate the necessity of adapting ARP conditions to accommodate the variable carbonate levels in natural and treated waters.

## 4 Conclusions

This study demonstrates that employing far-UVC irradiation at 222 nm in combination with sulfite-based advanced reduction processes significantly enhances PFAS degradation compared to conventional UV254-based approaches. The UV222/sulfite ARP generates higher yields of hydrated electrons, the primary reactive species for PFAS destruction, leading to more effective contaminant removal. A stepwise dosing of a moderate concentration (∼ 1 mM) of sulfite maintains a steady sulfite concentration and a high hydrated electron yield, leading to efficient degradation of PFAS by the UV222/sulfite ARP. Specifically, the UV222 system achieved removal efficiency as high as 85% for parent compound and 66% defluorination for PFOS within a 6 h treatment period. Furthermore, the UV222-based system also demonstrated notably improved energy efficiency, characterized by lower electrical energy per order compared to conventional UV254-based systems. This enhanced energy efficiency not only reduces operational costs but also aligns closely with sustainable environmental management goals, making it a more attractive approach than the conventional LPUV light sources. Common water matrix components such as nitrate/nitrite, DOM, and dissolved carbonates can influence performance by light-shielding and/or scavenging reactive species. Among them, nitrate/nitrite are consumed quickly under UV222, and then their inhibitory effect is reduced. Hence, understanding and managing water matrix interference is crucial to applying UV222/sulfite ARPs effectively under practical conditions. Overall, by demonstrating enhanced contaminant removal and energy efficiency, the use of far-UVC 222 nm irradiation in UV/sulfite ARPs presents a significant advancement in sustainable environmental remediation technologies, providing a viable and effective strategy for addressing the challenge of PFAS contamination in the environment.

## Supplementary Material



## Notes

Text S1, chemicals; (Text S2–S4) calculations of *R*_*e‑,UV*_, [e_aq_^–^], effective quantum yield and electrical energy per order; Table S1, PFAS analytes and MRM parameters; Tables S2, S3, S5, and S6, kinetic data of sulfite, MCAA and PFAS decay; Table S4, *E*_*EO*_ data; Figure S1, experimental setup; Figure S2, PFOS decay at different wavelengths; Figure S3, degradation products and proposed reaction mechanism; Figure S4, decay of nitrate/nitrite over time (PDF)
