# Supplementary material for: Far-UVC (222 nm) Enhances the Advanced Reduction Process for Per- and Polyfluoroalkyl Substance (PFAS) Destruction
Source: ACS ES T Water. 2025 Sep 12;5(10):6048–57. doi: 10.1021/acsestwater.5c00730 (PMC12519484; doi:10.1021/acsestwater.5c00730)
Supplement: Supplementary file 1 [file ew5c00730_si_001.pdf]

Supporting Information

for

Far-UVC (222-nm) Enhances the Advanced Reduction Process for Per- and Polyfluoroalkyl Substances (PFAS) Destruction

Xiaoyue Xin, Jiaqi Li and Ching-Hua Huang*

School of Civil and Environmental Engineering, Georgia Institute of Technology, Atlanta, GA 30332, United States

*Corresponding Author. Email: ching-hua.huang@ce.gatech.edu (Ching-Hua Huang)

Number of Pages: 17

Number of Texts: 4

Number of Tables: 6

Number of Figures: 4

Number of References: 7

Contents

Texts

Text S1. Chemicals and reaction solution preparation. --S3

Text S2. Calculation of $R_{e-,UV}$ and [e_{aq}^-]. --S4

Text S3. Calculation of effective quantum yield of UV/sulfite systems. ------------------------------------S6

Text S4. Calculation of electrical energy per order (E_{EO}). --S7

Tables

Table S1. PFAS analytes, and MRM acquisition mode parameters. ---S8

Table S2. Sulfite decay rate. ---S9

Table S3. MCAA decay rate and calculated effective quantum yield. --------------------------------------S9

Table S4. E_{EO} of UV/sulfite ARP systems under 222-nm and 254-nm irradiation. ---------------------S10

Table S5. Experimental results for selected PFAS decay and defluorination by UV/sulfite systems. --S11

Table S6. PFAS kinetic data. ---S12

Figures

Figure S1. Illustration of experimental set-ups. --S13

Figure S2. PFOS degradation by UV/sulfite systems at different irradiation wavelengths (222-nm and 254-nm) but on similar fluence basis. ---S14

Figure S3. (a) Peak areas of detected degradation products (intermediates) from PFOS; (b) Proposed reaction mechanisms for PFSA by UV222/sulfite ARP -------------------------------------S15

Figure S4. Decay of nitrate/nitrite as a function of time. --S16

Text S1. Chemicals and Solution Preparation.

Sodium sulfite (≥98%; Sigma-Aldrich), sodium tetraborate decahydrate (crystalline/certified ACS; Fisher Chemical), sodium nitrate (≥99.0%; Sigma-Aldrich), humic acid (≥90%; MP Biochemicals), potassium iodide (≥99.0%; Sigma-Aldrich), potassium iodate (99.5%; Sigma-Aldrich), sodium hydroxide (97%; Thermo Fisher Scientific), and hydrochloric acid (35%, Thermo Fisher Scientific) were used to prepare reaction solutions. LC-MS grade methanol (Fisher Optima), LC-MS grade acetonitrile (Fisher Optima), acetic acid (Fisher Optima), and LC-MS grade ammonium acetate (Fisher Optima) were used to prepare LC eluents. Dionex seven anion standard II (Thermo Fisher Scientific), and Dionex AS14A eluent concentrate (sodium carbonate/bicarbonate concentrate; Thermo Fisher Scientific) were used to prepare IC standards and eluents.

All PFAS individual chemicals were purchased from Sigma-Aldrich with the highest purity. All individual PFAS compounds (except for PFHxS) were dissolved in Milli-Q water to make 1.0 mM stock solutions. Due to its low water solubility, individual PFHxS was dissolved in 1.0 mM borate buffer (adjusted to pH 10) to prepare a 1.0 mM stock solution. All PFAS stock solutions were stored in 50 mL polypropylene (PP) tubes at 4°C.

The PFAS standards and isotope-labeled perfluorooctane sulfonate (PFOS) internal standard (sodium perfluoro-1[$^{13}C_8$]octanesulfonate) for LC- triple quad MS analysis were all purchased from Wellington Laboratories (Guelph, Canada).

Text S2. Calculation of $R_{e\text{-},UV}$ and [e_{aq}^-].

The hydrated electron exposure per UV fluence ($R_{e\text{-},UV}$) in the UV/sulfite ARP systems in this study was experimentally determined using the rate law for the loss of MCAA.[1]

$$\frac{d[MCAA]}{dt} = -\left(k_d' + k_{MCAA,\,e_{aq}^-} \times \left[e_{aq}^-\right]\right)[MCAA] \quad (S1)$$

k_d' = the rate constant for MCAA loss by direct photolysis (s^{-1})

$k_{MCAA,\,e_{aq}^-}$ = the bimolecular rate constant of MCAA reaction with hydrated electron (1.0×10^9 $M^{-1} \cdot s^{-1}$).[2]

For UV254, MCAA experiences negligible direct photolysis, **eq. S1** can be simplified and integrated to form **eq. S2**:

$$\ln\left(\frac{[MCAA]_t}{[MCAA]_0}\right) = -k_{MCAA,\,e_{aq}^-} \times \int_0^t [e_{aq}^-]_t dt \quad (S2)$$

For UV222, there is non-neglectable direct photolysis of MCAA, **eq. S1** can be integrated to form **eq. S3**:

$$\ln\left(\frac{[MCAA]_t}{[MCAA]_0}\right) = -k_d' \times t - k_{MCAA,\,e_{aq}^-} \times \int_0^t [e_{aq}^-]_t dt \quad (S3)$$

Dividing both sides of **eq. S2 and S3** by the UV fluence *H* (i.e., the fluence rate I_0 multiplied by the exposure time *t*) normalizes the e_{aq}^- exposure to the input UV fluence and yields $R_{e\text{-},UV}$ ($M \cdot s \cdot L \cdot Einstein^{-1}$):

$$H = I_0 \times \quad (S4)$$

For UV254:

$$R_{e^-,UV254} = -\frac{\ln\left(\frac{[MCAA]_t}{[MCAA]_0}\right)}{I_0 \times t \times k_{MCAA,\,e_{aq}^-}} = \frac{\int_0^t [e_{aq}^-]_t dt}{H} \quad (S5)$$

For UV222:

$$R_{e^-,UV222} = -\left(\frac{\ln\left(\frac{[MCAA]_t}{[MCAA]_0}\right) + k_d' \times t}{I_0 \times t \times k_{MCAA,\,e_{aq}^-}}\right) = \frac{\int_0^t [e_{aq}^-]_t dt}{H} \quad (S6)$$

Plot $\ln\left(\frac{[MCAA]_t}{[MCAA]_0}\right)$ against *t* to obtain the first-order rate constant k_{obs} (s^{-1}):

$$k_{obs} = -slope = -\frac{\ln\left(\frac{[MCAA]_t}{[MCAA]_0}\right)}{t} \quad (S7)$$

For UV254:

$$R_{e^-,UV254} = -\frac{\ln\left(\frac{[MCAA]_t}{[MCAA]_0}\right)}{I_0 \times t \times k_{MCAA,\,e_{aq}^-}} = \frac{k_{obs}}{I_0 \times k_{MCAA,\,e_{aq}^-}} = \frac{\int_0^t [e_{aq}^-]_t dt}{H} \quad (S8)$$

For UV222:

$$R_{e^-,UV222} = -\left(\frac{\ln\left(\frac{[MCAA]_t}{[MCAA]_0}\right) + k_d' \times t}{I_0 \times t \times k_{MCAA,\, e_{aq}^-}}\right) = \frac{k_{obs} - k_d'}{I_0 \times k_{MCAA,\, e_{aq}^-}} = \frac{\int_0^t [e_{aq}^-]_t dt}{H} (S9)$$

If $[e_{aq}^-]$ was assumed to be appreciably stable over the interval where the loss of MCAA was monitored, then the steady-state concentration of e_{aq}^- (M) generated in the UV/sulfite systems can be estimated by **eq. S10**:

$$[e_{aq}^-]_{ss} = R_{e^-,UV} \times I_0 (S10)$$

For UV222/sulfite systems, the contribution of MCAA direct photolysis decreased with increasing sulfite dosage. Hence, the corresponding rate constants for MCAA loss by direct photolysis (k_d') were determined using the following equations:

$$k_d' \times C_{t,MCAA} = -\frac{dC_{t,MCAA}}{dt} = \Phi_{MCAA,222} \times I \times \left(\frac{A}{V}\right) \times F_s \times F_c (S11)$$

I = incident light intensity at a certain wavelength (222 nm)
F_s = fraction of light absorbed by the system
F_c = fraction of light absorbed by the photoreactive substance

$$F_s = 1 - 10^{-(\alpha + \varepsilon_{SO32-} \cdot C_{t,SO32-}) \cdot l} (S12)$$

$$F_c = \frac{\varepsilon_{MCAA} \cdot C_{t,MCAA}}{\alpha + \varepsilon_{SO32-} \cdot C_{t,SO32-}} (S13)$$

α = the absorption coefficient of the solvent
ε = the molar absorption coefficient of the photoreactive substance
l = the light path length
eq. S11 can then be simplified to form **eq. S14**:

$$k_d' = \Phi_{MCAA,222} \times I \times \left(\frac{A}{V}\right) \times \varepsilon_{MCAA} \times \frac{1 - 10^{-\varepsilon_{SO32-} \cdot C_{t,SO32-} \cdot l}}{\varepsilon_{SO32-} \cdot C_{t,SO32-}} (S14)$$

The quantum yield of MCAA photolysis under 222-nm irradiation ($\Phi_{MCAA,222}$) without the presence of sulfite was determined using **eq. S15** to be 1.42 mol·Ein^{-1}:

$$\Phi_{MCAA,222} = \frac{k_d}{2.303 \times I \times \left(\frac{A}{V}\right) \times \varepsilon_{MCAA} \cdot l} (S15)$$

where the pseudo first-order rate constant k_d was experimentally determined through direct photolysis of MCAA under 222-nm irradiation without sulfite ((1.90 ± 0.12) × 10^{-4} s^{-1}).

Text S3. Calculation of effective quantum yield of UV/sulfite systems.

The effective quantum efficiency (Φ_{obs}, mol·Ein^{-1}) of the UV/sulfite system is defined as the moles of e_{aq}^- formed divided by the moles of photons absorbed by sulfite and can be calculated using **eq. S16**.[3]

$$\Phi_{obs} = \frac{r_0 V}{I(1-10^{-\varepsilon Cl})} (S16)$$

r_0 = the formation rate of e_{aq}^- (M·s^{-1}); it was assumed that all the formed e_{aq}^- participated in the reductive reactions with MCAA under strong alkaline conditions. Consequently, the degradation rates of MCAA could probe r_0.

V = solution volume (L)

I = the photon flux entering the solution (einstein·s^{-1})

ε = the molar absorption coefficient of SO_3^{2-} (M^{-1}·cm^{-1})

C = the concentration of SO_3^{2-}

l = the effective path length (cm)

For UV222/sulfite systems, the contribution of MCAA direct photolysis was nonnegligible and decreased with increasing sulfite dosage. The corresponding rate for MCAA loss by direct photolysis (r_d) were determined using **eq. S11**. Thus, r_0 in **eq. S16** were obtained by subtracting the direct photolysis rate of MCAA from the observed rate: $r_0 = r_{obs} - r_d$.

The effective quantum yield reported in this study was averaged values of quantum yields calculated based on overall MCAA decay rate under different sulfite dosages. The averaged quantum yield value was similar to the values calculated based on MCAA decay rate and averaged sulfite concentrations within every 60 s considering the variation of concentration of sulfite overtime (**Table S2**). Calculation results are summarized in **Table S3**.

Text S4. Calculation of electrical energy per order (E_{EO}).

The E_{EO} (kW·h·m^{-3}·order^{-1}) is defined as the number of kW·h of electrical energy required to reduce the concentration of the target compound by one order of magnitude (90%) in one m^3 of water. In this study, E_{EO} values of UV222 and UV254 ARP systems were estimated based on degradation of MCAA, representing hydrated electron generation efficiency. It consists of two parts, namely, the electrical energy of the UV lamp ($E_{EO,\,UV}$) and the equivalent electrical energy for reductant (i.e., sodium sulfite) consumption ($E_{EO,\,sulfite}$):

$$E_{EO} = E_{EO,\,UV} + E_{EO,\,sulfite} (S17)$$

$E_{EO,UV}$ can be calculated from the following equations:[4, 5]

$$E_{EO,\,UV} = \frac{1000 \times P \times t}{V \times \log\left(\frac{C_0}{C_t}\right)} (S18)$$

$$\ln\left(\frac{C_0}{C_t}\right) = k \times (S19)$$

where P is the rated power (kW) of the UV/sulfite ARP system, t is the irradiation time (min), V is the solution volume (L) in the reactor, C_0 and C_t are the initial concentration and concentration after irradiation time of t, respectively; k is the pseudo first-order rate constant (min^{-1}) for the decay of the target compound;

From **eq. S18** and **S19**, $E_{EO,UV}$ can be written as follows:

$$E_{EO,UV} = \frac{38.4 \times P}{V \times k} (S20)$$

$$P = \frac{I_e}{\eta} (S21)$$

where 38.4 is the constant resulting from the conversion between log vs ln and the unit conversion between min^{-1} and h^{-1}; I_e represents the UV irradiance on the solution surface (converted to kW); η represents the electric current wall-plug efficiency of lamps. One mole of photons (one Einstein) is equivalent to 0.1308 kWh of energy at 254 nm, and 0.1498 kWh of energy at 222 nm, respectively;[5] the current wall-plug efficiency of LPUV lamp is about 30–38% (35% used for calculation), KrCl* excimer lamps is about 5–15% (10% used for calculation), respectively.[6, 7]

$E_{EO,sulfite}$ can be calculated from the following equations:

$$E_{EO,\,sulfite} = E_{eq} \times \frac{1000 \times \left[SO_3^{2-}\right]}{\log\left(\frac{C_0}{C_t}\right)} (S22)$$

$$E_{eq} = \frac{Price \times Mass}{Purity \times EC} (S23)$$

where E_{eq} is the electric energy consumption generated per mole of sodium sulfite equivalently (kW·h·mol^{-1}). There is no reported value for sodium sulfite yet. *Price* represents the average price of sodium sulfite (608 USD/ton from imarcgroup.com); *Mass* represents the molar mass of sodium sulfite (126 g·mol^{-1}); *Purity* means the purity of sodium sulfite (~98% for industrial grade); EC means the average electricity cost in USA (17 cent/kWh from chooseenergy.com). E_{eq} in this study was thus calculated to be 0.46 kW·h·mol^{-1}.

Table S1. PFAS analytes and their MRM acquisition mode parameters.

**Analyte**	**Precursor Ion (m/z)**	**Product Ion(s) (m/z)**	**Retention Time (min)**
PFBS	298.7	79.9 / 98.9	7.4
PFHxS	398.7	79.9 / 98.9	8.3
PFOS	498.9	79.9 / 98.9	9.5
[$^{13}C_8$] PFOS	506.8	79.9	9.5

Table S2. Sulfite decay rate (k_s, min^{-1}) in UV/sulfite ARP systems under 222-nm and 254-nm. Reaction condition: $[MCAA]_0$ = 45 μM, $[SO_3^{2-}]_0$ = 0.5–10 mM, [borate] = 1 mM, pH = 10.

UV irradiation (nm)	Sulfite dose (mM)	***ks*** **(min^{-1})**
222	0.5	$(4.10 \pm 0.16) \times 10^{-2}$
	0.7	$(3.74 \pm 0.18) \times 10^{-2}$
	1.0	$(3.68 \pm 0.24) \times 10^{-2}$
	2.5	$(2.67 \pm 0.17) \times 10^{-2}$
	5.0	$(1.70 \pm 0.05) \times 10^{-2}$
	10.0	$(9.28 \pm 0.07) \times 10^{-3}$
254	1.0	$(8.23 \pm 0.24) \times 10^{-3}$
	2.5	$(5.59 \pm 0.43) \times 10^{-3}$
	5.0	$(5.64 \pm 0.27) \times 10^{-3}$
	10.0	$(3.00 \pm 0.08) \times 10^{-3}$

Table S3. MCAA decay rate (r, $M \cdot s^{-1}$) and calculated effective quantum yield (Φ_{obs}, $mol \cdot Ein^{-1}$) in UV/sulfite ARP systems under 222-nm and 254-nm. Reaction condition: $[MCAA]_0$ = 45 μM, $[SO_3^{2-}]_0$ = 0.5–10 mM, [borate] = 1 mM, pH = 10.

UV irradiation (nm)	Sulfite dose (mM)	r_{obs} (MCAA) ($M \cdot s^{-1}$)	*r_0 (MCAA) ($M \cdot s^{-1}$)	Ave Φ_{obs} ($mol \cdot Ein^{-1}$)
222	0.5	6.54×10^{-8}	6.38×10^{-8}	0.133
	0.7	7.25×10^{-8}	7.14×10^{-8}	
	1.0	9.51×10^{-8}	9.46×10^{-8}	
	2.5	8.07×10^{-8}	8.05×10^{-8}	
	5.0	7.16×10^{-8}	7.15×10^{-8}	
	10.0	5.77×10^{-8}	5.76×10^{-8}	
254	1.0	4.55×10^{-9}	/	0.129
	2.5	1.03×10^{-8}	/	
	5.0	1.82×10^{-8}	/	
	10.0	2.83×10^{-8}	/	

* r_0 (MCAA) for UV222/sulfite systems were obtained by subtracting the direct photolysis rate of MCAA from the observed rate: $r_0 = r_{obs} - r_d$.

Table S4. E_{EO} (kW·h·m^{-3}·order^{-1}) of UV/sulfite ARP systems under 222-nm and 254-nm irradiation. Reaction condition: V = 50 mL, t = 5 min, $[MCAA]_0$ = 45 μM, $[SO_3^{2-}]_0$ = 0.5–10 mM, [borate] = 1 mM, pH = 10, solution was initially purged by N_2 gas for 1 h.

UV irradiation (nm)	Sulfite dose (mM)	$E_{EO,UV}$ (kW·h·m^{-3}·order^{-1})	$E_{EO,sulfite}$ (kW·h·m^{-3}·order^{-1})	E_{EO} (kW·h·m^{-3}·order^{-1})
222	0.5	0.51	0.038	0.55
	0.7	0.47	0.049	0.52
	1.0	0.39	0.032	0.42
	2.5	0.42	0.090	0.51
	5.0	0.50	0.246	0.74
	10.0	0.82	0.883	1.71
254	1.0	0.90	0.454	1.35
	2.5	0.45	0.547	0.99
	5.0	0.36	0.865	1.23
	10.0	0.33	1.514	1.84

Table S5. Experimental results for selected PFAS decay and defluorination by UV/sulfite systems under different reaction conditions. Default reaction condition: $[PFAS]_0$ = 8 μM, [borate] = 1 mM, pH = 10, solution was initially purged by N_2 gas for 1 h, 4 h reaction time under 222-nm irradiation.

**PFAS**	**Change in reaction condition**	**Decay (%)**	**DeF (%)**
Comparison of PFOS decomposition under 254-nm and 222-nm irradiation			
PFOS	UV222, $[SO_3^{2-}]_0$ = 10 mM, 2 h	(41.3 ± 1.0)%	(26.2 ± 0.6)%
PFOS	UV254, $[SO_3^{2-}]_0$ = 10 mM, 3.5 h	(35.8 ± 3.9)%	(18.8 ± 2.1)%
Decay and defluorination of selected PFAS under different doses of sulfite (multiple portions)			
PFOS	$[SO_3^{2-}]_t$ = 0.5 mM	(38.4 ± 1.2)%	(21.2 ± 0.8)%
PFOS	$[SO_3^{2-}]_t$ = 0.7 mM	(47.1 ± 1.8)%	(23.1 ± 0.8)%
PFOS	$[SO_3^{2-}]_t$ = 1.0 mM	(71.8 ± 1.5)%	(49.8 ± 5.7)%
PFOS	$[SO_3^{2-}]_t$ = 2.5 mM	(71.8 ± 6.8)%	(44.3 ± 5.8)%
PFOS	$[SO_3^{2-}]_t$ = 5.0 mM	(64.2 ± 3.7)%	(41.3 ± 1.8)%
PFOS	$[SO_3^{2-}]_t$ = 10.0 mM	(59.0 ± 3.9)%	(31.2 ± 1.6)%
*****Decay and defluorination of selected PFAS***			
PFOS	6 h	(85.2 ± 1.0)%	(66.4 ± 2.8)%
PFHxS	10 h	(73.2 ± 0.4)%	(33.5 ± 2.4)%
PFBS	10 h	(18.4 ± 1.3)%	(8.1 ± 0.7)%
*****Effect of real water matrix***			
PFOS	Ideal borate buffer	(85.2 ± 1.0)%	(66.4 ± 2.8)%
PFOS	$[nitrate]_0$ = 2 mg·L^{-1}	(90.5 ± 1.5)%	(54.1 ± 4.3)%
PFOS	$[HA]_0$ = 1 mg·L^{-1}	(51.3 ± 5.2)%	(28.5 ± 3.6)%
PFOS	$[HA]_0$ = 10 mg·L^{-1}	(34.3 ± 7.3)%	(19.0 ± 4.4)%
PFOS	$[carbonate]_0$ = 1 mM	(58.3 ± 5.5)%	(31.3 ± 3.2)%
PFOS	$[carbonate]_0$ = 5 mM	(40.1 ± 5.1)%	(15.7 ± 2.1)%

**Optimized reaction condition: $[PFAS]_0$ = 8 μM, $[SO_3^{2-}]_t$ = 1 mM (sulfite was spiked into waters at time 0 and added every hour to maintain a concentration of around 1 mM), [borate] = 1 mM, pH = 10, solution was initially purged by N_2 gas for 1 h, 6 h reaction time under 222-nm irradiation.

Table S6. PFAS kinetic data: pseudo first-order reaction rate constant ($k_{PFAS,obs}$) obtained from PFAS decay under optimized reaction condition (this study); bimolecular rate constants ($k_{PFAS,eaq-}$) obtained from steady-state under optimized reaction condition (this study), describing selected PFAS reacting with e_{aq}^-. Optimized reaction condition: $[PFAS]_0$ = 8 μM, $[SO_3^{2-}]_t$ = 1 mM (sulfite was spiked into waters at time 0 and added every hour to maintain a concentration of around 1 mM), [borate] = 1 mM, pH = 10, solution was initially purged by N_2 gas for 1 h, reaction under 222-nm irradiation.

**PFAS**	$k_{PFAS,obs}$ **(s^{-1})**	$k_{PFAS,eaq-}$ **($M^{-1}\cdot s^{-1}$)**
PFOS	$(9.08 \pm 0.12) \times 10^{-5}$	$(1.83 \pm 0.02) \times 10^{7}$
PFHxS	$(3.33 \pm 0.07) \times 10^{-5}$	$(6.71 \pm 0.01) \times 10^{6}$
PFBS	$(5.99 \pm 0.22) \times 10^{-6}$	$(1.21 \pm 0.04) \times 10^{6}$

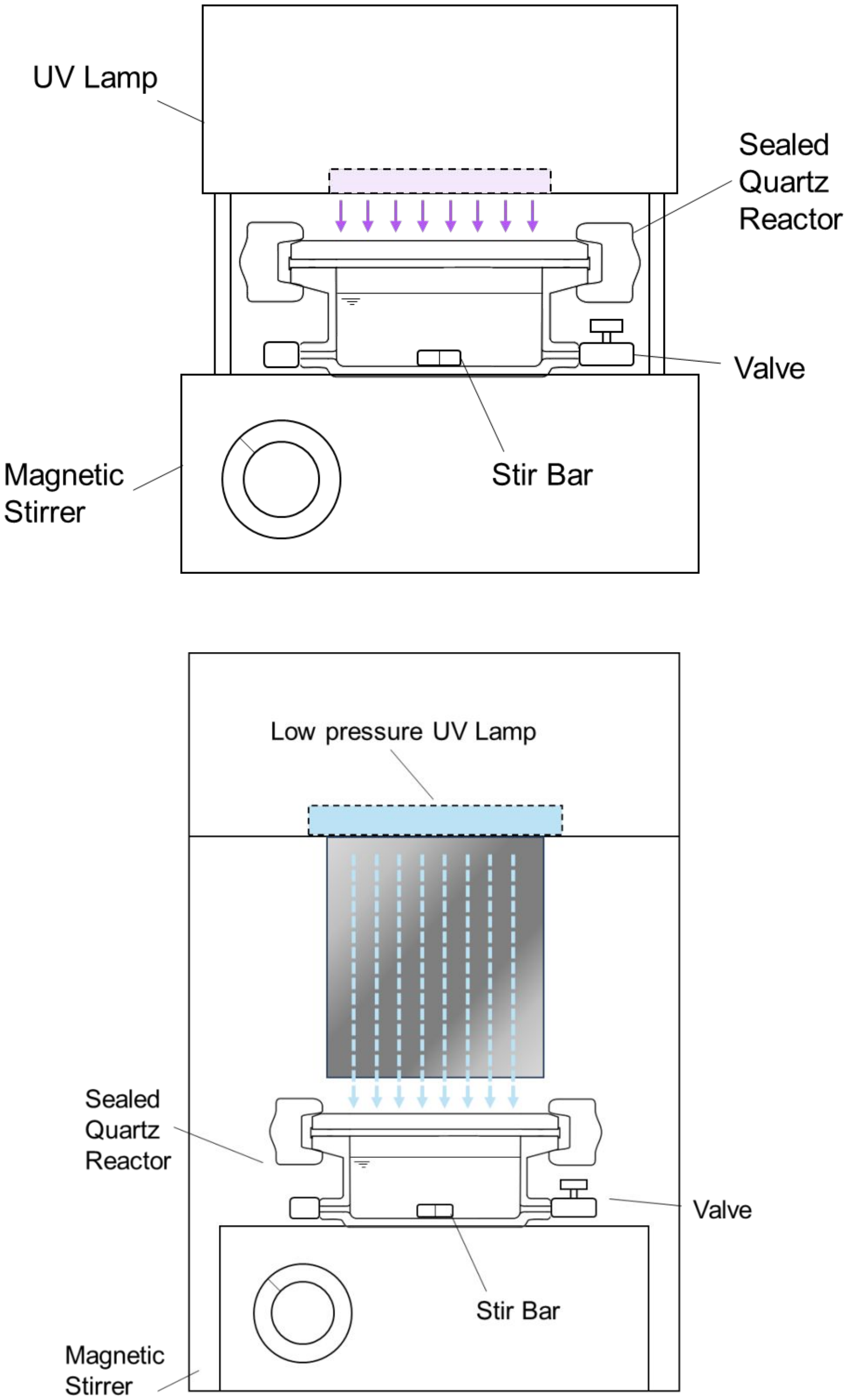

Figure S1. Illustration of experimental set-up using 222-nm KrCl* excimer lamp (top) and 254-nm LPUV mercury lamp (bottom).

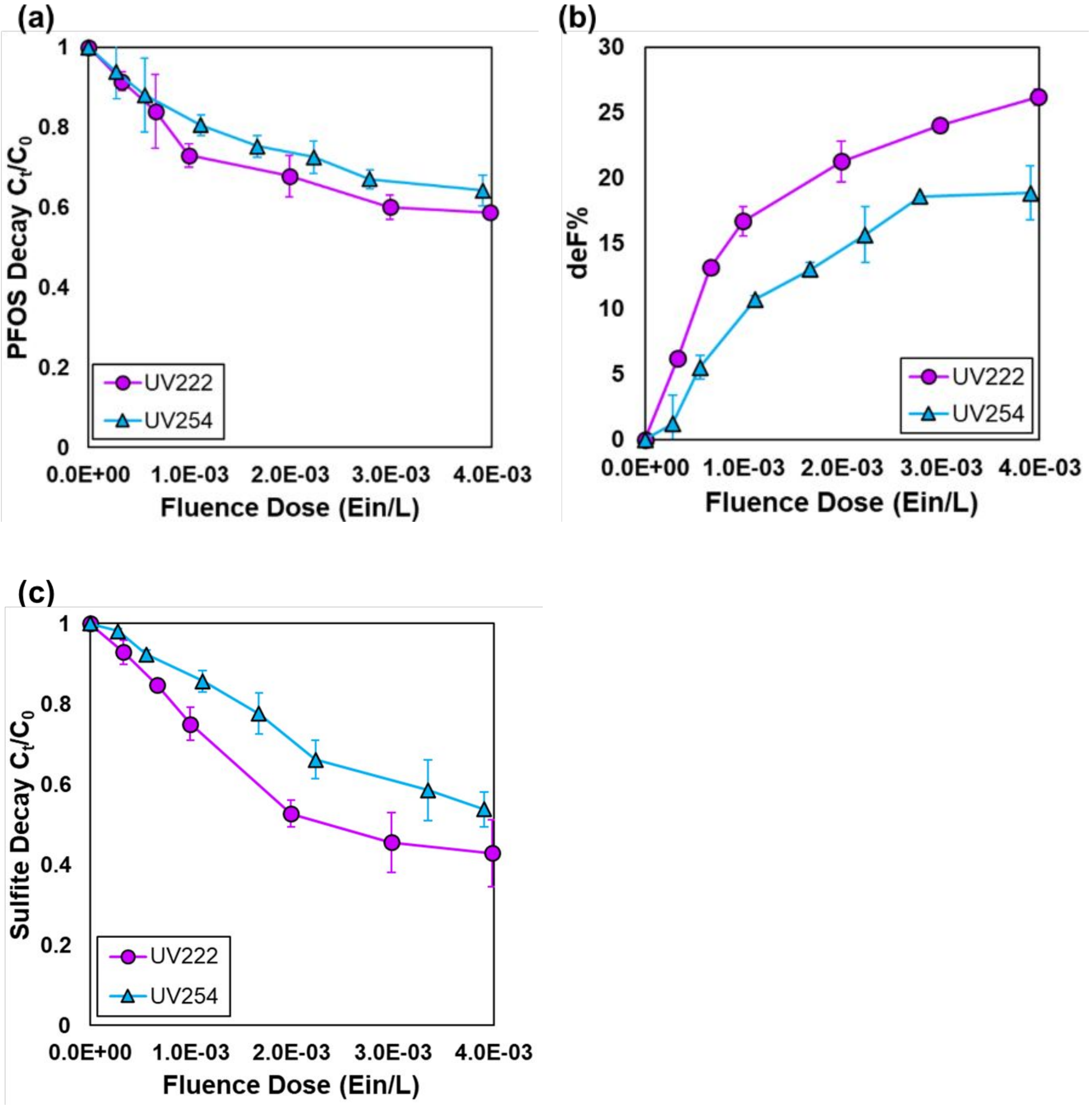

Figure S2. PFOS degradation by UV/sulfite systems at different irradiation wavelengths (222-nm and 254-nm) but on similar fluence basis (~4.0×10^{-3} Einstein·L^{-1}). (a) PFOS decay; (b) PFOS defluorination; (c) sulfite decay over time. Reaction conditions: $[PFOS]_0 = 8$ μM, $[SO_3^{2-}]_0 = 10.0$ mM, [borate] = 1.0 mM, pH = 10.

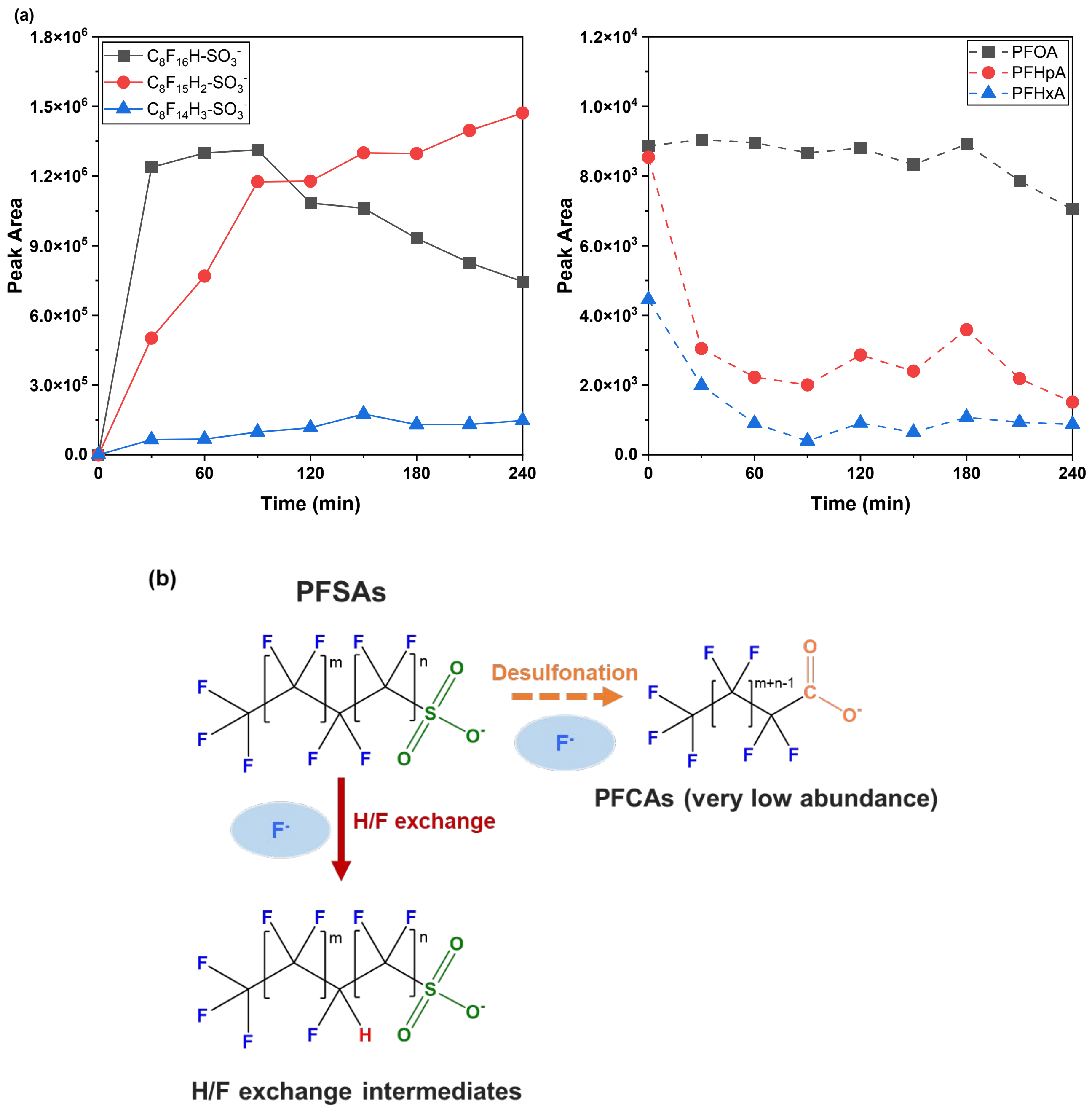

Figure S3. (a) Peak areas of detected degradation products (intermediates) from PFOS within 4-h by UV222/sulfite ARP. Reaction condition: $[PFOS]_0$ = 8 μM, [borate] = 1.0 mM, pH = 10, sulfite was spiked into water at time 0 and added every hour to maintain a concentration of around 1 mM. (b) Proposed reaction mechanisms for PFSA degradation and defluorination by UV222/sulfite ARP.

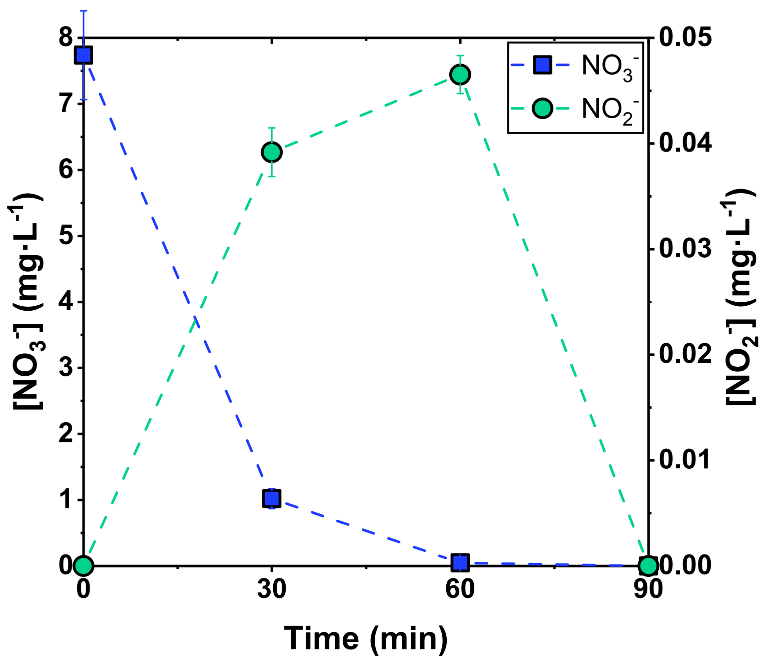

Figure S4. Decay of nitrate/nitrite as a function of time. Reaction conditions: $[PFOS]_0$ = 8 μM, [borate] = 1.0 mM, pH = 10, $[nitrate]_0$ = 2 mg·L^{-1} (as N); sulfite was spiked into waters at time 0 and added every hour to maintain a concentration of around 1 mM.

References

1. Fennell, B. D.; Odorisio, A.; McKay, G., Quantifying Hydrated Electron Transformation Kinetics in UV-Advanced Reduction Processes Using the R_{e-}, UV Method. *Environ. Sci. Technol.* **2022,** *56*, (14), 10329-10338.
2. Buxton, G. V.; Greenstock, C. L.; Phillips Helman, W.; Ross, A. B., Critical review of rate constants for reacitons of hydrated electrons. *J. Phys. Chem. Ref. Data;(United States)* **1988,** *17*, (2).
3. Li, X.; Ma, J.; Liu, G.; Fang, J.; Yue, S.; Guan, Y.; Chen, L.; Liu, X., Efficient reductive dechlorination of monochloroacetic acid by sulfite/UV process. *Environ. Sci. Technol.* **2012,** *46*, (13), 7342-7349.
4. Cater, S. R.; Stefan, M. I.; Bolton, J. R.; Safarzadeh-Amiri, A., UV/H_2O_2 treatment of methyl tert-butyl ether in contaminated waters. *Environ. Sci. Technol.* **2000,** *34*, (4), 659-662.
5. Bolton, J. R.; Bircher, K. G.; Tumas, W.; Tolman, C. A., Figures-of-merit for the technical development and application of advanced oxidation technologies for both electric-and solar-driven systems (IUPAC Technical Report). *Pure Appl. Chem.* **2001,** *73*, (4), 627-637.
6. Yang, T.; Zeng, G.; Jiang, M.; Su, P.; Liu, C.; Lv, Q.; Li, W.; Hou, X.; Li, J., Matching periodate peak absorbance by far UVC at 222 nm promotes the degradation of micropollutants and energy efficiency. *J. Hazard. Mater.* **2024,** *476*, 134978.
7. Kim, J.; Xin, X.; Kann, R. J.; Li, J.; Labrozzi, A. S.; Xu, J.; Huang, C.-H., Photodegradation of Nitrogenous Disinfection Byproducts by Far-UVC Light at 222 nm. *ACS EST Water* **2025,** *5*, (5), 2619-2629.
